# Stakeholder Focus Groups to Inform a Technology-Based Strategy of Preceptor Support

**DOI:** 10.1155/2012/246532

**Published:** 2012-05-07

**Authors:** Cynthia A. Blum, Jeanette Kamciyan, Amy Dean

**Affiliations:** ^1^Christine E. Lynn College of Nursing, Florida Atlantic University, 777 Glades Road, Boca Raton, FL 33431, USA; ^2^Nursing Education, Bethesda Memorial Hospital, 2815 S. Seacrest Boulevard, Boynton Beach, FL 33435, USA; ^3^Grants Department, Palm Healthcare Foundation, 1016 North Dixie Highway, West Palm Beach, FL 33401-3306, USA

## Abstract

While preceptors are a vital link in student nurse practice education, ongoing support beyond an initial orientation is often lacking. It has been reported in the literature that preceptors experience stress related to difficulties in handling preceptee situations. They are frustrated by negative experiences centered on preceptor-identified hallmarks of unsafe practice including the inability to demonstrate knowledge and skills; attitude problems; unprofessional behavior; and poor communication skills. Their unrealized expectations for novices threaten their commitment to their preceptor role. As part of a larger study testing the effectiveness of podcasts as an ongoing method of preceptor support, this paper addresses the developmental stage of the podcasts. A team of academic and acute care nurse educators developed scripts for eventual filming of four podcasts focusing on unsafe practice issues, designed to provide continual support through web-based availability. The use of podcast technology is consistent with the learning styles of digital natives and is a demonstrated and valuable educational resource to review, reinforce, and clarify difficult concepts. These podcasts were informed through preceptor focus groups to address situational and environmental realism for student behaviors and preceptor responses.

## 1. Introduction

A preceptor serves as an expert in his or her field and provides practical experience and training to a student or novice. The central role nurse preceptors play in supporting the transition of student nurses and new nurses into professional practice is well recognized. Yet, ongoing support for nurse preceptors is too often lacking in clinical settings [1–4]. Staff nurse preceptors expect support from their practice site [5, 6]. Although support is initially provided, it frequently diminishes or ends after orientation [1–4, 7]. Preceptors view support as recognition from the institution, authorization to share from their personal experiences, and the opportunity to create environments for safe practice through mentoring students and novice nurses [8]. Realizing these layers of preceptor support, a model has been proposed that guides precepting and mentoring of students and novice nurses by infusing caring behaviors to address areas of unsafe practice noted by preceptors. Podcasts created to address unsafe practices are expected to serve as an innovative educational mechanism for providing support consistent with the usual learning practices for staff nurse preceptors, most of whom are digitally competent. This podcast approach to ongoing support provides an opportunity to strengthen and build the preceptor workforce, concurrently reinforcing caring connections between staff and students or novices. The findings of this research have the potential to provide guidance to nurse leaders regarding innovative ways to prepare and support nurse preceptors.

### 1.1. Background

Since 2002, Palm Healthcare Foundation, Inc., a grantmaking philanthropy serving Palm Beach County, Florida, has been convening the Healthcare Workforce Partnership (HWP) to advance professional nursing practice. Today, the HWP is a volunteer community collaboration of nurse leaders and nursing stakeholders from a variety of healthcare settings and academe that serves as a catalyst for positive change; a resource to ensure an adequate supply of highly educated and trained nursing practitioners and faculty; and fosters innovation in the study and practice of nursing. The success of the HWP is grounded in the diversity of its members; a spirit of openness and collegiality by all the partners; and the ability to leverage and share resources and expertise across the community.

Over the years, the members of the HWP have identified specific strategies for, and implemented a number of programs and initiatives designed to address shared nursing challenges, including the nursing shortage; nursing leadership development; and the transition to professional practice for new nurses. Formal strategic planning sessions and a committee structure—including an education-practice gap (EPG) committee—facilitate this work.

Recognizing the critical role preceptors play in developing nurse professionals and the bridge they provide between the academic and clinical settings, the EPG committee has focused its work on efforts to strengthen the skill-set and role commitment of these key educators. This work builds on earlier initiatives of the HWP, including a *Preceptor of Excellence* training and recognition program that the partnership conducted from 2004 to 2008 for approximately 450 nurse preceptors throughout Palm Beach County. In addition, a series of focus groups and surveys conducted by the EPG committee in 2008 and 2009 with nurse preceptors and preceptees identified the need to expand involvement and cultivate the preceptor-student relationship.

Strengthening the preceptorship experience for students and preceptors supports needed realignment and change in nursing education, as detailed in the recent Institute of Medicine [9] report, “*The Future of Nursing: Leading Change, Advancing Health*”, and The Carnegie Foundation for the Advancement of Teaching study, “*Educating Nurses: A Call for Radical Transformation*” [10]. The velocity of healthcare system and practice change and the growing knowledge demands for nurses are challenging academia to keep pace [11]. As nursing education is redesigned to produce an adequate number of well-prepared nurses to meet current and future health care demands [9], preceptor support and lifelong learning opportunities are critical because preceptors not only are an immediate link to student, but also represent potential future nurse faculty.

### 1.2. Literature Review

Nursing knowledge learned in the classroom comes alive when experienced staff nurse preceptors share their current practice-based knowledge with novice nurses [12, 13]. Preceptors have honed their teaching skills through patient teaching and can be expected to transfer this ability into teaching others in the clinical setting [13]. In a comprehensive literature review, Billay and Yonge [14] identify common defining preceptor attributes as role modeling, facilitating, communicating, sharing knowledge, and using adult education principles. Preceptors are accountable for providing bedside teaching and competency evaluation of novice nurses [15], ideally in a one-to-one relationship [13, 16] designed to facilitate novice nurse role development. The relationships between the nurse and novice epitomize caring, as an expression of the humanness of other [17].

According to Carper, “Caring is not readily, if at all, learned in a classroom or a formal course of study” [18, page 18]. Rather, “Caring is developed within the practitioner while practicing nursing, by modeling others and learning by doing, and may be demonstrated without intention, and without the language to express the actions that follow” [19, page 29]. The relationship between the staff nurse and novice thrives in the context of caring where each honors the other's gifts [17], but infusion of caring demands structural guidance for staff nurse preceptors engaged with novice nurses.

Preceptors expect support from their place of employment [5, 6], as well as from affiliated academic institutions [20–22]. Most frequently, support is provided as a formal, face-to-face preceptor orientation in preparation for coaching novice nurses [5, 23], although web-based preceptor courses have also been successful [24]. After orientation, updates in preceptor knowledge may be provided, but ongoing preceptor support is less likely to occur [1–4]. Once a preceptor is “established,” that person is considered the resident resource for novices within the organization. Preceptors often report stress related to the difficulties in handling preceptee situations [7, 25, 26]; they are frustrated by negative experiences centered on preceptor-identified hallmarks of unsafe practice including: the inability to demonstrate knowledge and skills; attitude problems; unprofessional behavior; and poor communication skills [27]. Their unrealized expectations for novices threaten their commitment to their preceptor role [28].

The *expectation gap *between the preceptor and novice can make the difference in retaining practicing nurses, new hires, and the potential employee that a novice represents [7, 21]. The frustrated and overworked staff nurse of today may be hesitant to take on the responsibility of educating a novice nurse unless he or she recognizes available support systems and resources [20, 29]. Podcasts can be a supportive resource to address preceptor frustrations. The use of podcasts is a novel way to incorporate technology [30], consistent with the learning styles of digital natives. Podcasts serve as a demonstrated and valuable educational resource to review, reinforce, and clarify difficult concepts [31, 32]. Convenience and accessibility enable the preceptor to utilize this resource immediately when support and knowledge are needed most (reactively), or at any time when learning is sought (proactively).

## 2. Materials and Methods

This study was designed to develop and test educational podcasts that infuse caring principles into situations of unsafe practice that concern staff nurse preceptors. For the purpose of this study, preceptees were presented as student nurses, although the researchers acknowledge the larger role that preceptors assume with all novice nurses. Consistency of student nurses as preceptees was chosen to avoid confusion during initial development and testing of the podcasts.

The podcasts provide an opportunity for the preceptor to learn effective ways of dealing with challenging situations, serving as a mechanism of ongoing support with potential to increase commitment to the preceptor role. This paper addresses the development of the podcasts as informed by qualitative focus groups. Resultant podcast scenarios will serve as a tool to assist the preceptor to use a caring approach when dealing with situations identified as unsafe [27].

Podcast scripts were written by the EPG committee based on the four hallmarks of unsafe practice [27]. Caring attributes of preceptors as identified through thematic analysis of BSN student interviews were incorporated in the scripts including; welcoming presence, demonstrating empathy, encouraging growth, patience and time as compassionate care, building relationships, and communicating therapeutically [19]. Small subgroups of two to three committee members were responsible for initial script development within a scenario, each addressing one of these hallmarks. The scripts were further refined through email exchange of ideas and a committee meeting. Preceptor caring responses were purposively embedded in the detailed scripted scenarios. The scenarios were then reviewed by Dr. Anne Boykin, an expert on caring theory, who agreed to be a consultant for this work. Dr. Boykin worked with the committee to assure the infusion of caring principles in the developed podcast prior to review by the focus group participants.

The intent of the qualitative focus groups was to have a range of informants provide feedback on the realism and credibility of each of the four podcast scripts. The committee felt it was important to include the stakeholders in review of the final podcast scripts, prior to filming taking place. Two small focus groups were held; this strategy is consistent with the recommendation of Morgan [33] to employ a smaller group when the participants are experts or well acquainted with the topic.

Initially, the researchers specified a central location to hold the focus groups, but the limited response prompted them to hold the focus groups at two designated locations that were convenient to the participants—one of which was a hospital and the other a college of nursing. Nurses responded voluntarily to e-mails, announcements from instructors and committee members, and/or conversations with one of the researchers to discuss their inclusion. No additional points or grades were attached to these activities and non-participation was not penalized. Participants were given a $20 gift card in appreciation of their time and expertise that was shared.

Two focus groups with different participants were held one week apart; each of the focus groups reviewed all four podcasts scripts. The first focus group lasted for 60 minutes and the second lasted for 70 minutes, including completion of the associated university IRB approved informed consent and demographic information. All focus groups were recorded and transcribed; field notes were written, and content was analyzed consistent with recommendations by Krueger [34, 35].

### 2.1. Participants

 Each focus group consisted of five or six participants. The final sample size of 11 provided a purposive convenience sample of practicing nurses; a total of ten females and one male with an average age of 33 years (range = 20–59 years). Participants reported from less than one year to 26 years of nursing experience (mean = 4.821), and zero to six years preceptor experience (mean = 0.95). The participants' experience with various types of preceptees was reported as zero to five occasions precepting newly hired nurses; zero to two occasions precepting nurses transferring from another department; zero to 15 occasions precepting nursing students; and no experience precepting but self-described recent experience as a preceptee. The mean total number of precepting experiences was 3.27, with a range from zero to ten. Only 18% of the sample reported engaging in preceptor training of any kind. An Associate of Arts or Associate of Science was the highest educational degree earned for 55% of the participants, 36% were Baccalaureate of Science or Baccalaureate of Science in Nursing graduates, and 9% were Master of Science or Master of Science in Nursing degree graduates.

### 2.2. Focus Group Procedures

 After explaining the purpose of the focus group research and obtaining informed consent from the participants, the principal investigator (PI) guided the focus group interview. First, the PI asked the participants to read each script (Table 1 summarizes the final content of the podcast scripts).

After each script was read, the PI asked three questions designed to direct the focus group discussion: Is this script true to nurse preceptor experiences? Are the nursing responses appropriate? and What would you add or remove from the scenario to enhance realism?

## 3. Results

The focus group interviews were recorded and transcribed. Field notes were written and used in the analysis process. Focus group content analysis was consistent with the Krueger method [35]. Inductive processes were used to identify themes, culminating in both generalized and specific guidance to enhance podcast realism and meaning for practice. Field notes were also considered which allowed the researchers to note key points, notable quotes, and observe body language and inflection, providing valuable insight. No identifying information was used in the analysis of transcriptions. The researchers read transcripts and field observations independently. Using descriptive analysis, six themes emerged in response to the key questions, which guided revision of the podcast scripts.

 The first key question the PI asked the focus group participants was whether the scripts were true to nurse preceptor experiences. Transcript analysis and field note consideration identified the following themes.

**Modeling and Learning Professionalism**

Professionalism is a key concern of nurse preceptors, which was thought to be best learned through modeling of professional behaviors. The participants felt that within the scripts, the preceptor's tolerance of a student's actions and attitudes was not always consistent with nursing practice. Furthermore, they felt that in some scripted situations, the preceptors were too permissive and allowed students to inappropriately take control. They did not feel that those behaviors would help the student learn professionalism, and they commented that the preceptor should be a model for appropriate behaviors. An example of these behaviors was stated as follows:

“To me, I think the preceptor is awfully friendly, a lot friendlier than I have ever encountered ever, and a lot more time is spent understanding the student. I mean it's awfully nice, but it's not realistic.”

Some participants expressed differences between past personal experiences as preceptee conflicting with the preceptor caring behaviors represented in the scripts. The PI informed the participants that although the scripted interactions may not reflect their own precepting experiences, the purpose of the podcasts was to model caring behaviors critical to successful precepting.

*Defining reality* is a theme that identified truthfulness to nurse preceptor experiences in actual practice situations, a commonly identified division between nursing education idealism and nursing practice realities. When reviewing the scripts, there was some controversy about the reality of practice versus academic instruction. For example, in a script in which medications were administered there was discussion regarding whether the practicing nurse would ask patients the purpose of their medications (potentially preventing an error), or whether the nurse would inform the patient of the purpose of the medications while administering them. Students are taught to first ask the patient in order to assess knowledge but the preceptors were divided on this point; some stating that instruction while giving medications is more time efficient and others feeling it best to clarify what patients state they have learned about the medications. Overall, it was felt that either method is a variant of practice that is equally acceptable. Moreover, utilizing either method would not detract from the fact that the preceptor handled the prevention of the potential error professionally, as stated below:

“She didn't, you know, like correct the student like right in front of the patient, let the patient know that there was an error going to happen, embarrass the student you know, none of that, she handled it very professionally.”

Several participants thought that with the advent of medication administration software, many medication errors would be prevented in the future. But, the reality of nurse preceptor experiences may be in conflict with how students are taught.

The second key question the PI asked the preceptor focus groups was whether the nursing responses were appropriate. From the raw data, descriptive themes emerged.

**Finding Solutions**

Nurse preceptors feel caught between their responsibility as preceptors, and knowing how to handle uncomfortable student situations. At times, academe lacks clear guidance on the preceptor's role. The participants felt that the preceptor should consistently demand professional behaviors from the student, who needed to set aside personal problems and focus on the patient. They felt that there should be repercussions if a student is not prepared for an assignment, such as a drop in grade, allowing the student to observe without hands-on nursing care, or getting a third party involved, such as the manager of the unit or the instructor. Most of the participants agreed that, the majority of the time, the student should not be sent home because it would be punitive and counter-productive. Instead, they suggested that if the preceptor stated the expectations for the student's performance ahead of time, it might keep the student focused.

In addition the group suggested other potential solutions:

“…Maybe not being able to pass meds at all for the day or just observation, because as students we really want to get the hands-on practice unless her goals are not in the right place. But, usually, we really want to get involved and get some hands-on knowledge. So maybe by telling her, “well, since you're not prepared you cannot pass any medications today”; and usually as a student she will be very upset. Like, oh man, so I better make sure I am prepared next time.”

Preceptors and preceptees learn from each other. The theme* Working and learning together *describes how focus group participants felt the scripts could be enhanced, by showing the student performing patient care and demonstrating how scripted interactions between the preceptor and student enhanced the student's ability. After the demonstration, the preceptor and student could have a dialogue to demonstrate confidence in the student's ability, the value of teamwork, and a reminder that the preceptor will always be available for student support as stated here:

“When the student nurse mentioned like, I just hope I don't forget anything,… I would give them like a reaffirming statement like, “you're not in this on your own”, or,…, Yes, I do trust you enough to do this on your own; if you need my help or support I am still here for you.”

More specifically, the focus group participants felt that the scripts should not present intentionally conflicting situations; such as both patients' medications being pulled at the same time, particularly with sound-alike/look-alike medications. However, the participants said it is realistic that you would pick more than one patient's medication from the PYXIS at one time. Some participants in this group thought that the student should be given more independence while having the support of the preceptor as a safety net, which provided an excellent learning opportunity as stated here:

“And that was good she let her go through the motions up until the point you know, but it's good. I think she was kind of waiting to see if maybe she would catch her own mistake. I think it was handled well.”

In this example the nurse preceptor and student were *working and learning together* and the nursing responses were deemed appropriate.

The final key focus group question “What would you add or remove from the scenario to enhance realism?” triggered specific suggestions from the focus group participants to “fine-tune” each of the podcast scripts. The participants shared the importance of *setting realistic expectations*, an overarching theme throughout this work. In one script, the participants felt that the preceptor should have reviewed the discharge instructions with the student before going to the patient's room, to be sure that the student was prepared and knew the important points to review with the patient. Since there are too many things at stake, including core measures and patient satisfaction, the participants said that the preceptor would never let the student give discharge instructions alone:

“But if we teach them and we set the tone, “this is what we expect from you” instead of let them do whichever way they come from, this way we will first prevent and make sure that they do the right thing….”

*Reproducing reality* is the final theme related to the appropriateness of nursing responses as identified by the focus group participants. This theme differs from *defining reality *in that “defining” identifies education versus practice realities, where “reproducing” refers to the realities of contemporary society. For example, in one script the student was talking on a cell phone while in a patient room. While the participants agreed that the preceptor handled the cell phone situation very professionally (explaining not only that the behavior was unprofessional, but also that it was against the rules, unsafe, and why), the participants suggested that the scripted unprofessional behavior change from a phone call to texting, to reflect contemporary concerns as stated below:

“I can see how they would, the student here would think, oh, it's you know, because we pick up our *Spectralinks* ^®^in the room. … But, I think texting would be better.”

## 4. Discussion

 The focus group participants identified many different concerns to guide revision of the podcast scripts, thereby allowing the potential stakeholders to inform this technology-based intervention of ongoing preceptor support. Each of the podcast scripts was revised to reflect the suggestions gleaned through thematic analysis of focus group content. Podcast (1) *attitude problems*—was made less wordy, but struck a better balance between the intolerance for the student's lack of preparedness and the preceptor's sensitivity to the student's attitude. Indeed, this scenario provoked the most discussion between reviewers of this podcast. The focus groups' feedback served to enhance the dialogue and the intended outcome. Podcast (2) *communication*—was revised to include the student providing the discharge instructions to the patient a second time, after the preceptor and student had the opportunity to discuss discrepancies with the student's method and content. The student performance expectations were emphasized in the revised script, as well as preceptor support and confidence in the student's ability. Minor revisions were made to Podcast (3) *inability to demonstrate knowledge*—since the focus group participants agreed to the realism of the situation, particularly with senior level students. In addition, the focus group participants concurred that, although software may mitigate medication errors, they still might occur and near misses proved an invaluable learning tool. The script was revised to emphasize that the preceptor brought the look-alike sound-alike phenomena to the student's attention before an error could be made. In the script for Podcast (4) *unprofessional behavior*—the phone call was changed to a texting scenario, in keeping with the unanimous suggestion from both focus groups. The focus group feedback aided the researchers in creating a final product with enriched content authenticity and authority, which was the overall intent of the focus group approach.

Although the intent of the study was to include only experienced preceptors in the focus groups, three of the participants recruited by an EPG committee member were novice nurses who had not served as preceptors, but had recently been preceptees. Initially, it was considered that this might limit the development of the scripted scenarios. In actuality, these participants brought personal insights to the student role, which created lively discussion within this group. These recent preceptees shared that, although the situations may be realistic, the scripted preceptor's actions were not consistent with some of their personal experiences. Surprising to the researchers, the recent preceptee participants were more likely to suggest sending the student preceptee home or to take punitive actions. However, they also appreciated the kinder, gentler approach to potentially unsafe preceptee behaviors suggested in the scripts. They discussed how reviewing these scripted podcast scenarios would make them less likely to repeat inappropriate preceptor behaviors that were experienced in their personal student development.

The focus group participants were enthusiastic about the availability of the podcasts to serve as an ongoing resource for nurse preceptors. In addition to responding to the focus group questions for the podcast scripts addressing previously identified hallmarks of unsafe practice [25], the participants offered the following suggestions for future podcast themes:

- teamwork, interacting with coworkers;
- communicating with physicians;
- computerized physician order entry;
- incorporation of core measures into the scenarios.

## 5. Conclusions

Involving the stakeholders when designing a technology-based strategy of preceptor support has enhanced this project and conjoined nursing education and nursing practice. The focus group participants supported the infusion of technology-based podcasts as a method of ongoing preceptor support. Finally, the participants, representing a range from recent preceptees to experienced preceptors, brought practical experience to the design of the podcast scripts by answering the questions; Is this script true to nurse preceptor experiences? Are the nursing responses appropriate? and What would you add or remove from the scenario to enhance realism?

### 5.1. Limitations

 A possible limitation of this study may have arisen when novices and experienced preceptors were comingled within one focus group. Krueger [35] warns that focus group participants may influence each other, sometimes seeking to convert an individual's point of view. While intimidation from either group was not observed in body language or discussions, this does not preclude a member's reluctance to share an opinion regarding one of the scripted scenarios. However, having gained the unexpected insight from the novice nurses regarding their experience with preceptorships, perhaps another stakeholder group, that of students, will be added to elicit feedback on future podcasts from their perspective. An important lesson learned from our unintentional inclusion of novice nurses in the focus groups is that all stakeholders need to inform the podcasts, which emerges from their unique vantage point as to the realism of the situations.

### 5.2. Future Directions

Content saturation occurred quickly with the focus groups and the participants supported further development of this project. The podcasts have been filmed and are currently being edited. The next step of this funded study is to make the podcasts available to a selected group of preceptors during their experience with students and evaluate their perception of support using this technology-based intervention. Those research results will direct the further development of a *library* of podcasts that would be continuously available and without cost to all preceptors via a publically accessible website. While student nurses have served as the preceptee within the initial scripted scenario, further podcasts might represent preceptor engagement with novice nurses.

Future directions of this work are to research the effectiveness of podcasts as teaching tools to improve preceptor skills, facilitate knowledge transfer to preceptees, and improve the quality of patient care. As nurse residency programs continue to emerge and the central role that preceptors inhabit is acknowledged, podcast technology has the potential to develop and support preceptors in a cost-effective and contemporary manner.

## Figures and Tables

**Table 1:** Educational podcast design.

Preceptor identified hallmarks of unsafe practice [27]	Summary of podcasts
Attitude problems	The nursing student seems unprepared, unfocused, and overwhelmed, and just wants to “put in her hours” as opposed to seeking a quality experience. The nurse preceptor creates a supportive atmosphere as she helps the nursing student identify and work through her difficulties.
Poor communication skills	This podcast focuses on professional communication between student and patient, centering on the delivery of discharge instructions. The nurse preceptor has an opportunity to observe the student's approach and create a supportive atmosphere as she reviews critical aspects of this responsibility with the nursing student.
Inability to demonstrate knowledge and skills	A senior level nursing student is preparing to administer medication to two patients. The nursing student mixes up the medications for the two patients. The nurse preceptor gently stops the student and corrects the mistake before the patient takes the medication. Together the preceptor and student discuss what occurred (outside of the patient's room) and how to prevent a reoccurrence.
Unprofessional behavior	While interacting with a patient during medication administration, the nursing student is texting on her personal cell phone. The nurse preceptor politely excuses both himself and the student and discusses the situation privately with the student in a supportive and constructive manner.
